# Is expanding Medicare coverage cost-effective?

**DOI:** 10.1186/1472-6963-5-23

**Published:** 2005-03-14

**Authors:** Peter Franks, Peter Muennig, Marthe Gold

**Affiliations:** 1 Center for Health Services Research in Primary Care, Department of Family & Community Medicine, University of California, Davis, Sacramento, CA, USA; 2 Department of Health Policy and Management, Mailman School of Public Health, Columbia University, New York, NY, USA; 3 City University of New York Medical School, New York, NY, USA

## Abstract

**Background:**

Proposals to expand Medicare coverage tend to be expensive, but the value of services purchased is not known. This study evaluates the efficiency of the average private supplemental insurance plan for Medicare recipients.

**Methods:**

Data from the National Health Interview Survey, the National Death Index, and the Medical Expenditure Panel Survey were analyzed to estimate the costs, changes in life expectancy, and health-related quality of life gains associated with providing private supplemental insurance coverage for Medicare beneficiaries. Model inputs included socio-demographic, health, and health behavior characteristics.

Parameter estimates from regression models were used to predict quality-adjusted life years (QALYs) and costs associated with private supplemental insurance relative to Medicare only. Markov decision analysis modeling was then employed to calculate incremental cost-effectiveness ratios.

**Results:**

Medicare supplemental insurance is associated with increased health care utilization, but the additional costs associated with this utilization are offset by gains in quality-adjusted life expectancy. The incremental cost-effectiveness of private supplemental insurance is approximately $24,000 per QALY gained relative to Medicare alone.

**Conclusion:**

Supplemental insurance for Medicare beneficiaries is a good value, with an incremental cost-effectiveness ratio comparable to medical interventions commonly deemed worthwhile.

## Background

Medicare, the national health insurance program for the elderly in the U.S., consists of various plans or "parts [1]". Part A covers hospitalization costs and has no premium, but limits and deductibles apply. Part B covers outpatient care and requires a small monthly fee as well as a deductible. Limited prescription drug coverage will be added in 2006, but medications, co-payments, and deductible costs presently create a large and growing private market for Medicare supplemental insurance [2]. Retirees without access to supplemental insurance as a work-related benefit may choose to purchase Medicare supplemental insurance out-of-pocket, a phenomenon that may increase in the near future [3]. However, about 15% of elderly persons forego private supplemental insurance policies, a number that may increase as the cost of policies increase.

There is evidence that elderly persons who lack private supplemental insurance may be at increased risk of morbidity and mortality [4,5]. Approximately 35% of the near poor elderly lacking supplemental insurance either skip doses or do not fill their prescriptions at all because they cannot afford the medications they are prescribed. Medicare supplemental insurance may improve access to care by reducing co-payments and medication costs, thereby reducing barriers to timely care for acute but treatable illnesses, chronic disease care, as well as preventive services. Among younger cohorts, co-payments have been shown to reduce utilization and possibly increase mortality [6-9]. Though improved access to medical care appears to confer health benefits, there is considerable uncertainty as to whether these benefits are worth their cost.

For the government, sensible decision-making requires an understanding of the health benefits associated with purchasing additional services. Much of the debate about the Medicare Modernization Act of 2003 has focused on its overall cost in the face of large deficiencies in coverage [2,10]. With more employers likely to withdraw from carrying retiree costs past the age of Medicare eligibility, and the costs of insurance spiraling, greater numbers of older Americans are likely to be underinsured. Despite a rapidly growing budget, policymakers will likely be asked to consider improving the package that Medicare offers.

Given the uncertainty surrounding the efficiency of Medicare supplemental insurance however, policymakers have little rational basis upon which to design future Medicare policy changes. Information on the cost-effectiveness of the additional medical goods and services provided by a supplemental policy to Medicare would provide a foundation for evaluating Medicare reform initiatives. It also has implications for estimating the cost-effectiveness of universal coverage proposals. To these ends, we conducted a cost-effectiveness analysis of the average private supplemental insurance policy relative to Medicare alone. Such an analysis, together with sensitivity analyses of key parameters, may provide a benchmark with which to assess the efficiency of other services and technologies that vie for addition to the Medicare market basket.

Previous studies have examined the costs associated with providing health insurance to younger cohorts [11]. Other studies have examined the effectiveness of health insurance in general [6-9]. In this study, we use a natural history Markov model to estimate the cost-effectiveness of supplemental medical insurance among the elderly, updating and amalgamating these earlier studies. Our cohort consists of persons over the age of 65 who presently do or do not have insurance.

## Methods

### Overview and definitions

To estimate the expenditures and health effects of private supplemental Medicare insurance to persons over age 65 relative to those who lack such coverage, we used a 3-step process. First, we performed regression analyses on nationally representative data of Medicare recipients with private supplemental insurance. These analyses modeled the effects of socio-demographic, behavioral and clinical variables on expenditures, health-related quality of life (HRQL) values, and mortality.

Those without supplemental insurance coverage differ in socio-demographic characteristics from those that do have coverage. We therefore apply the socio-demographic characteristics of the uninsured to regression models of insured persons. The use of the predictive functions of linear regression analysis allows for a better estimate of the impact of providing supplemental insurance to the uninsured.

Finally, using Markov decision analysis modeling that incorporates the predicted costs and health benefits, we estimated the cost-effectiveness of providing private supplemental insurance to the average 65-year-old with Medicare only coverage.

We use the quality-adjusted life year (QALY) as an outcome measure. The QALY combines health-related quality of life with life expectancy; one QALY represents a year of life lived in perfect health [12,13]. In our analysis, the incremental cost per QALY gained is the additional money spent on supplemental health insurance divided by the additional gains in quality-adjusted life expectancy. This ratio is called the incremental cost-effectiveness ratio (ICER). Our study was conducted from the societal perspective, employed a community derived measure of HRQL to calculate effectiveness in terms of QALYs, and used a 3% discount rate, following recommendations of the Panel on Cost-Effectiveness in Health and Medicine [12]. We did not capture over-the counter drug costs, costs associated with institutionalized persons, or direct non-medical costs.

Health-related quality of life scores were derived from the Health and Activities Limitation Index (HALex) [14]. The HALex is comprised of two health domains, self-rated health and role limitations. The HALex exhibits reasonable validity, but because it captures only 2 health domains, its sensitivity to the full spectrum of morbidity is limited [15,16]. Details of these methods have been published elsewhere [14].

The most recent publicly available nationally representative information on the relationships between health insurance and HRQL and mortality is the 1993 National Health Interview Survey (NHIS) linked to the 1995 National Death Index [17,18]. We therefore used these data to the to obtain prospective HRQL and mortality differentials by private supplemental insurance coverage status. We obtained costs from the 1996 Medical Expenditure Panel Survey (MEPS) [11,19].

### Regression analyses

We developed regression models using SUDAAN (Research Triangle Park, NC) and STATA (College Station, TX), adjusting for the complex sample designs used in both NHIS and MEPS. In these models, HRQL scores, survival, or total medical expenditures were entered as dependent variables. Age, race, ethnicity, gender, education, family income (NHIS) or percent of federal poverty level (MEPS), family size, marital status, behavioral risk factors (smoking status and seatbelt use in NHIS), number of conditions reported (NHIS) or self-rated health (MEPS), and area of residence (region of country, and urban or rural location) were entered as independent variables. In the expenditure models (MEPS), only persons whose insurance status did not change throughout the year were included.

We estimated the effects of the socio-demographic and health variables upon HRQL scores using linear regression. Expenditures were analyzed using generalized linear regression models with a gamma distribution and a log link function (to account for the skewed distribution of expenditures). The resulting parameter estimates from the analyses using those with private supplemental insurance were used to calculate predicted HRQL scores and expenditures for those with Medicare only using their socio-demographic, behavioral and health characteristics. The predicted expenditures were adjusted to include administrative costs for health insurance plans. All costs were deflated to constant 1994 dollars using the medical portion of the consumer price index.

Proportional hazard models were used to estimate the risk of death due to lacking private supplemental health insurance.

### Decision analysis models and sensitivity analyses

We examined these costs and benefits over the lifetime of the average 65-year-old using a Markov decision analysis model using DATA Professional 4.0 (TreeAge Software, Williamstown, MA). Our models evaluated two possible events: giving private supplemental insurance to those lacking it, or receiving only Medicare parts A and B. For each health state, subjects were exposed to an annual, age-specific risk of death, with survivors gaining one HRQL-adjusted year and medical costs. The values for these year-to-year changes in health by insurance status were obtained from our regression analyses. Thus, the average cost of medical care for a 65 year-old given private supplemental insurance is tabulated and the model is advanced one year. Age-specific mortality rates are used to determine the proportion of subjects dying, and survivors are assigned a discounted QALY and costs for the next year. This process is repeated until over 99% of the subjects are dead. The same process is applied to the Medicare only cohort, and incremental values are calculated.

The variables used in our analyses were subjected to a Monte Carlo simulation and 1-way sensitivity analyses. In a 1-way analysis, all variables are held constant but one. In a Monte Carlo simulation, values for all variables are randomly sampled from a statistical distribution [20]. In our Monte Carlo simulation, we used a triangular distribution. In this distribution, the base-case estimate is entered as the most likely value and values between the high and low value are linearly interpreted. The assumptions of the analyses are listed in Table 1.

**Table 1:** Principal assumptions of the analysis.

*Subjects will remain in stated insurance category*. We did not have information on whether subjects continuously or intermittently lacked private supplemental insurance in all datasets.
*Administrative costs associated with private supplemental insurance companies are the only relevant costs*. It was assumed that costs associated with provider and hospital administration of supplemental policies and employer administrative costs would be negligible.
*Private supplemental insurance company administrative costs are proportionate to expenditures*. We calculated administrative costs using an expenditure driven formula [see Additional file 1].
*We included all relevant covariates in our regression models*. It is possible that unmeasured variables explain the observed effects of insurance on costs, mortality, and HRQL.
*Expenditures included in the MEPS survey reflect all relevant costs*. The costs reported in the MEPS do not include the institutionalized population, over-the-counter (OTC) medications, or alternative care.

The decision analysis models were validated by comparing their outputs with abridged health-adjusted life tables we generated using spreadsheets [21]. Markov modeling allows for a more accurate estimation of year-to-year effects by accounting for the effect of mortality on cost and effectiveness. It also simplifies sensitivity analyses. Abridged life tables allow for clear and simple estimations of changes in life expectancy when different interventions are applied and may thus serve to validate Markov models.

Detailed descriptions of the data sources, regression models, and the decision analysis models are available [see Additional file 1].

## Results

Hazard ratio estimates were unstable when the behavioral risk factors were included since including them reduced the sample size. However, behavioral risk factors exerted little effect on differences in mortality by insurance status. For our base-case analysis, we therefore used the larger sample, excluding the behavioral risk factors, but reduced the obtained adjusted hazard ratios by 10% [see Additional file 1].

In 1996 expenditures for persons with private supplemental insurance were higher than for those without private supplemental insurance ($4915 vs. $3956). These higher expenditures are reflected in greater utilization and prescription use (Table 2).

**Table 2:** Relationship between private supplemental insurance, selected utilization, and expenditures in the 1996 Medical Expenditure Panel Survey.

	Medicare only	Private supplemental insurance
Physician office visits	6.7	8.3
Physician office visit expenditures	$415	$745
Prescriptions filled	16.5	19.4
Prescription expenditures	$565	$722
Out-of-pocket prescription expenditures	$419	$371
Insurance prescription expenditures	$6	$333

Because of their lower socioeconomic status and lower health status, the predicted medical expenditures among those provided private supplemental insurance coverage would be higher than those who currently have it (Table 3). The average cost of supplemental insurance coverage would also be slightly higher ($1349 vs. $1270). Our models also predict small improvements in HRQL, and relatively larger improvements in mortality, but both remain worse than those currently with private supplemental insurance (Table 3).

**Table 3:** Observed and predicted expenditures and health outcomes

Expenditures			
Age-group	Observed Private supplemental insurance	Observed Medicare only	Predicted*

65–74	$4029	$3632	$4141
>75	$5942	$4243	$5919
Health-related quality of life score			
65–74	0.78	0.71	0.73
>75	0.70	0.62	0.67
Mortality†			
65–74	0.043	0.067	0.048
>75	0.083	0.129	0.092

*Values for effects of supplemental insurance on those with Medicare only using predictive regression models.

† Proportion dying within 24 months.

Providing lifetime private supplemental insurance to the average 65-year-old would cost society an additional $22,000 and would produce a gain of 0.88 QALY – an incremental cost-effectiveness ratio (ICER) of $24,000 per QALY gained (Table 4).

**Table 4:** Results of cost-effectiveness analyses at a 3% discount rate.

Strategy	Cost (USD)	Incremental Cost	Effectiveness	Incremental Effectiveness*	Incremental Cost-Effectiveness†
Cost per quality-adjusted life year (QALY) Gained					
Medicare only	$34,000	-	10.06 QALYs	-	-
Supplemental	$56,000	$22,000	10.94 QALYs	0.88 QALYs	$24,000
Cost per life year (LY) gained					
Medicare only	$34,000		13.26 LYs		
Supplemental	$56,000	$22,000	14.14 LYs	0.88 LYs	$24,000

*Rounded to 2 decimal places.

†Rounded to nearest thousand dollars.

### Sensitivity and sub-analyses

Table 5 lists the results of one-way sensitivity analyses on ICERs. Over a plausible range of high and low values, predicted administrative costs exerted the greatest effect on the incremental cost-effectiveness of supplemental health insurance, resulting in a range of values from $21,000 per QALY gained to $41,000 per QALY gained. In the Monte Carlo analyses, 95% confidence interval of the ICER values for private supplemental insurance relative to Medicare only ranged from $12,000 to $36,000 per QALY gained. The Markov model and spreadsheet produced similar quality-adjusted life expectancy differentials between groups.

**Table 5:** Variables included in the decision analysis model, their assigned high, low, and baseline values, and the effect on the incremental cost-effectiveness ratio (ICER) of insurance relative to no insurance.

	Used in Model*			Effect on ICER	
Variable	Baseline	High	Low	High	Low

Administrative costs	5%	25%	0%	$41,000	$21,000
Hazard Ratio	1.5	1.75	1.25	$19,000	$37,000
Discount rate	3%	5%	0%	$26,000	$23,000
Error in cost†	0%	125%	0.75%	$39,000	10,000

* All values represent the percentage change in the baseline value except for the hazard ratio, which was varied from previously published estimates to the high value observed in logistic regression analysis [see Additional file 1].

†Variability in the cost estimate for the supplemental Medicare insurance. The baseline cost is multiplied by an error term ranging from 0.75 to 1.25.

**Figure 1:**
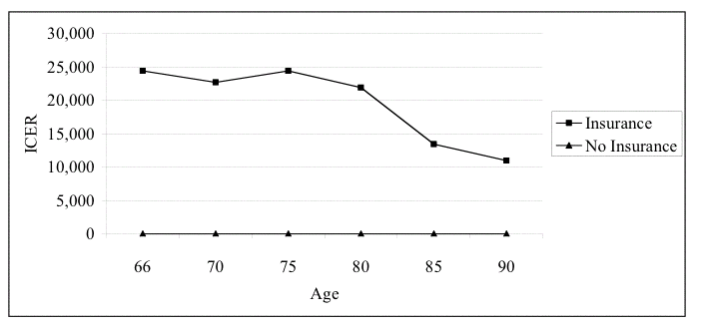
Changes in the incremental cost-effectiveness (ICER) of providing private supplemental insurance to those with Medicare at different ages.

Figure 1 shows the effects of providing private supplemental insurance at different ages. There is generally a trend toward increasing cost-effectiveness with age.

Finally, to estimate the effect of medical inflation under the assumption that health care becomes no more effective over time, we examined the ICER of private supplemental insurance in 2001 dollars. With effectiveness held constant, inflation has a differential effect of the cost of medical care for the insured relative to the uninsured, increasing the ICER to $30,000 per QALY gained.

## Discussion

Our models predict that the average private supplemental insurance plan provided to Medicare recipients is associated with substantial health benefits. Because health insurance increases the utilization of an array of medical goods and services, the impact of providing private supplemental insurance to the elderly is predicted to be relatively large, improving life expectancy in the population by about 11 months.

The ICER of private supplemental insurance is smaller than many medical interventions employed in day-to-day practice [22]. Moreover, incremental expenditures would be considerably lower than investments in many other well accepted social programs (e.g., airline and automobile safety) per life year gained [23].

The cost-effectiveness of supplemental insurance appears to increase with age (see Figure 1). This may reflect the increasing absolute net benefit of medical care interventions with increasing age; that is, as the absolute risk of mortality increases with most conditions with increasing age, so the potential absolute net benefit of treating those conditions also increases. The slight decrease in cost-effectiveness at age 70 is likely due to random error, as the sample sizes in each age-group become progressively smaller. Also, the mean expenditures of those in the sample in the age-group without supplemental insurance was notably higher than that of persons in older and younger age-groups.

Our analyses are susceptible to at least two main contravening biases. First, persons who choose to purchase private supplemental insurance may be more health oriented, and it is that orientation, not insurance, that is associated with improved outcomes. Second, and conversely, persons may purchase private supplemental insurance because they perceive themselves to be at higher health risk than is captured in the covariates we used. To control for health orientation, we included two behavioral risk factors, seatbelt use and smoking status, as covariates. However, risky health behavior can be quite nuanced. For example, there is evidence that persons with greater medical skepticism are less likely to have health insurance, have lower health care utilization, and have higher mortality [24,25]. Thus, the use of a cost-effectiveness ratio potentially mitigates some of these biases; those without supplemental insurance may have worse health habits and thus worse outcomes even if insured, but they may also be less likely to utilize health care even if they are given supplemental insurance – thereby generating lower costs than predicted [24,25]. It should be noted that in this single observational study one cannot be certain of the extent of the possible contravening biases, so that net bias may remain in the estimates used.

There are other notable limitations to our analyses. First, the data sources we used, though they are the most recent publicly available nationally representative samples, are somewhat dated. While the costs of medical care have outpaced general inflation, the effectiveness of medical interventions has also increased. The net effects of these changes are uncertain. However, even if effectiveness showed no improvement, the ratio would increase to just $30,000 per QALY gained in 2001 dollars.

Second, as suggested by Table 2, private supplemental insurance coverage is associated with both higher utilization of ambulatory care and greater prescription use. The analyses reflect the predicted impact of an average private supplemental insurance policy and do not provide information about the effects of specific components. The recent controversy over the decision to provide limited Medicare coverage for lung reduction surgery suggests that insurance coverage decisions will increasingly have to deal more explicitly with both the benefits and costs of specific components of health care [26].

Our analysis rests on two major assumptions. First, private supplemental insurance increases utilization of healthcare. Second, this increased utilization results in improved health. Interestingly, our data suggest that private supplemental insurance produces little improvement in health-related quality of life, with both incremental life expectancy and quality adjusted life expectancy rounding out to 0.88 (Table 4). It is unlikely that the risks of medical care among the living elderly (e.g., impotence secondary to anti-hypertensive medications) outweigh the benefits conferred by this care (e.g., reduced stroke due to anti-hypertensive medications). This is an area that requires further study.

Summarizing these limitations, we acknowledge that neither this study nor any individual study using observational data may adequately address the problems of endogeneity (in econometric terms) or confounding (in epidemiological terms) or establish causality. Additional studies are needed using different datasets and different approaches. Within the econometric framework, studies employing propensity scores or instrumental variables (if good instruments can be found) may yield less biased estimates of the effects described here. Within an epidemiological framework, studies using a broader array of potential confounders may also be helpful. Finally, other study designs employing quasi-experimental methods may be helpful: for example examining the effects of involuntary loss of insurance coverage, or comparing costs and outcomes for persons with more generous coverage provided as a result of prior employment (rather than current choice) to those with less generous coverage. Until such studies are available, the results reported here should be seen as providing a starting-point to examine the incremental cost-effectiveness of specific proposed additions to the overall Medicare package.

## Conclusion

Our analyses are not intended to suggest that expanding private supplemental health insurance plans is the most efficient means of improving health outcomes. A simple expansion of Medicare benefits targeted at covering medication costs and reducing inpatient co-payments may be less expensive. The General Accounting Office and Congressional Budget Office, for instance, has found that government programs may offer substantial savings in administrative costs over private sector plans [27,28]. Our analysis does suggest, however, that expanding supplemental health insurance for the elderly may be relatively cost-effective, with a cost-effectiveness ratio similar to that of younger cohorts [29].

## Competing interests

The author(s) declare that they have no competing interests.

## Authors' contributions

PF conducted the regression analyses and made major contributions to the development of the manuscript. PM conducted Markov and sensitivity analyses and made major contributions to the development of the manuscript. MG provided guidance for the study and contributed to the development of the manuscript.

## Pre-publication history

The pre-publication history for this paper can be accessed here:



## Supplementary Material

Additional File 1 Technical appendix. Is health insurance cost-effective? A technical appendix that describes methodology in more detail and provides additional data that might be of interest to readers. Click here for file
