# Supplementary material for: Is expanding Medicare coverage cost-effective?
Source: BMC Health Serv Res. 2005 Mar 14;5:23. doi: 10.1186/1472-6963-5-23 (PMC1079833; doi:10.1186/1472-6963-5-23)
Supplement: Additional File 1 — Technical appendix. Is health insurance cost-effective? A technical appendix that describes methodology in more detail and provides additional data that might be of interest to readers. [file 1472-6963-5-23-S1.pdf]

Technical Appendix

Is health insurance cost-effective?

Introduction

This technical appendix provides detailed information on the analyses used to examine the cost-effectiveness (CE) of health insurance. These analyses involved examining the CE of a) insuring persons 25-64 without insurance, and b) providing supplemental insurance to persons 65 and older who have Medicare only. Because the policy implications are different we present the analyses in separate papers. However, the details of the analyses underlying the papers are essentially identical. Thus, this technical appendix presents those details in one unified format. The sequence is: A) the methods and results for the analyses of the relationship between insurance status and both health-related quality of life and mortality; B) the methods and results for the analyses of the relationship between insurance status and expenditures; and C) the methods and results for the decision analyses

A: Insurance Status and Health-Related Quality of Life and Mortality (NHIS)

Methods

Sample

The 1993 NHIS included questions on health insurance status in the second half of the year, and included a sample of 61,287 persons. Of these, 38,500 were in the two study age groups, >25-64 and >65, of whom 37,185 had information allowing reliable mortality follow-up. The study samples excluded persons reporting they had no insurance because of poor health (59) or had lost their insurance because of unemployment (302). Also not included were persons with Medicaid, Medicare in persons < 65, those with military insurance, and those with other publicly funded

insurance programs. Thus, the samples included a) those 25-64 reporting either no insurance or private insurance (N=24,578); and b) those > 65 with Medicare only or private insurance as well (N=5,458). A subset of these NHIS respondents were also asked about their health risk behaviors, including smoking status and front seatbelt use. Information on these risk factors was available on 12,092 persons 25-64 and 3,294 persons >65.

Analyses

Two prediction models were developed for health status using the two insured cohorts. The dependent variables were the Health Activity Limitation Index (HALex) scores (also known as the Years of Healthy Life measure), and the independent variables were sociodemographic indicators, the number of conditions, and behavioral risk factors. A squared age term was included in each model to test for non-linearity, but no evidence of non-linearity was found. Predictors were excluded if they made no significant contribution ($p > .15$) to the models. The parameter estimates from these prediction equations were then applied to the respective variables for each person in the uninsured and Medicare only cohorts to yield a predicted HALex for each person.

Two Cox proportional hazard models were used to assess the adjusted contributions of insurance status in each cohort. Survival was assessed as the time in months between the interview date and the end of 1995, or the date of death, if sooner. The independent variables were insurance status, sociodemographic variables, health status, and behavioral risk factors. The models were tested for the violation of the proportional hazard assumption with regards to insurance status and no evidence of violation was found. The models also examined the interaction between insurance status and other key variables, specifically, age, number of conditions and employment status. To assess the potential confounding effect of the number of conditions a subject had at the time of the

interview, the analyses were run with this variable excluded and were then compared with the main models.

In the NHIS, questions pertaining to smoking status and seatbelt use were gathered on only a subset (< 50%) of the sample. As a result of the smaller sample size, and the relative infrequency of mortality in the 2-year follow-up, when behavioral risk factors were included in the proportional hazards models the model appeared to be overspecified. Thus the analyses were conducted first without the behavioral risk factors. The analyses were then conducted, excluding non-significant variables, and replacing categorical versions of the variables with modified continuous versions (to reduce the degrees of freedom). Smoking status was included as "ever smoker" or "never smoked,"[1] and seatbelt use was dichotomized as always/mostly vs. less often. These reduced versions of the models were then analyzed with the behavioral risk factors included and excluded to estimate the change in the parameter estimates for the insurance status variables, and thereby the extent of likely confounding.

Results

Table 1 shows the distribution of variables by insurance status. For those < 65, the uninsured are younger, have lower family income, are more likely to be male, have less education, are less likely to be white, less likely to be employed, more likely to live in the west and south, less likely to live in non-central MSA areas, more likely to be ever smokers , and less likely to use seatbelts. There was no difference in the number of conditions (mean 0.7 for both), but those with no insurance were in larger families (3.2 vs. 3.0). After multivariate adjustment, using logistic regression (Table 2), all these variables revealed similar independent associations with insurance status.

For those > 65 (Table 1), those without supplemental insurance are older, have lower incomes, more likely to be female, have less education, are less likely to be white, less likely to be working, more likely to be married, with a spouse in the home (and less likely to be widowed), more likely to live in the south and west, less likely to live in non-central MSA areas, less likely to be ever smokers, and less likely to use seatbelts. Those without supplemental insurance had more conditions (1.8 vs. 1.7) and larger families (2.2 vs. 1.8). After multivariate adjustment, using logistic regression (Table 2), the following variables remained statistically significant: income, gender, education, race/ethnicity, marital status, region, and seatbelt use.

Health status was lower in those without insurance (0.84 vs. 0.89), and those without supplemental insurance (0.67 vs. 0.75). Table 3 shows the results of the regressions of health status on covariates for those who were insured (<65) and had supplemental insurance (>65), and the parameter estimates used to derive the health status for the cohorts without insurance or supplemental insurance that would be predicted if they had insurance or supplemental insurance. Average health status for those without private insurance predicted if they obtained private insurance was 0.854 for those < 65, and 0.727 for those > 65.

After adjusting for covariates, mortality rates were obtained for the insured persons using the same age intervals. These rates were then multiplied by the hazard ratios for persons 25-64 and 65 and over to obtain rates specific to the uninsured cohort. Table 4 shows the actual and predicted health status by age group and associated mortality probabilities for these cohorts.

The relationships of insurance status with subsequent mortality adjusted for all variables excluding the behavioral risk factors are shown in Table 5. The adjusted hazard ratio (HR) for those < 65 was 1.73 (95% confidence interval (CI) = 1.10, 2.72) and for those > 65 was 1.56 (95% CI = 1.21, 2.02). The analyses were repeated including and excluding the behavioral risk factors,

using modified continuous or dichotomous variables and including only significant variables. Under 65 there were only 53 deaths in the sample, > 65 there were 224. Under 65, seat belt use showed no evidence of being associated with mortality. Over 65 both risk factor variables were associated with mortality, but there was equivocal evidence of confounding of the associations of insurance status with mortality in both age-groups. That is, the change in the HR when the behavioral risk factors were included or excluded was always less than 10%. For example, using continuous versions of the variables, and including only significant covariates other than insurance status and the behavioral risk factors, the HR for insurance for those < 65 was 1.70 (95% CI = 0.83, 3.50), it increased slightly if the behavioral risk factors were excluded to 1.72 (95% CI = 0.83, 3.56). For those > 65, the HR was 1.51 (95% CI = 1.06, 2.17) with the behavioral risk factors included and 1.54 (95% CI = 1.07, 2.21) with the behavioral risk factors excluded. Because of the uncertainties in the extent of confounding we reduced our base estimates of the HRs by 10% from the values observed in the full models excluding the behavioral risk factors. That is, we used a hazard ratio of 1.66 for persons < 65, and 1.49 for persons >65.

There was no statistical evidence of interaction between the insurance status variables and age, employment status, or number of conditions. There was little evidence of confounding of the insurance status effect by number conditions. When the number of conditions variable was excluded, the HR for insurance status changed relatively little to 1.72 (95% CI = 1.09, 2.70).

B: Insurance Status and Expenditures (MEPS)

Methods

Samples

The 1996 Medical Expenditure Panel Survey included information on 13,535 persons in the two age groups 25-64 and > 65. In our main study samples we included only those who reported no change in their insurance status during the 12 months of 1996. We compared a) for persons 25-64 those without insurance with those with private insurance, and b) for those > 65 those with Medicare only with those reporting private insurance also.

Analyses

Two prediction models were developed for total expenditures using the two insured cohorts. The dependent variables were total expenditures, and the independent variables each of the sociodemographic variables and self-rated health.[2] A squared age term was included in each model to test for non-linearity, but no evidence of non-linearity was found. The parameter estimates from these prediction equations were then applied to the respective variables for each person in the uninsured cohorts to yield a predicted total expenditure for each person.

Expenditures are not normally distributed, about 11% of persons under 65 and 3% of persons over 65 have no expenditures, while for those that have expenditures there is a significant right skewing of expenditures. A number of approaches have been proposed for dealing with these problems, and it is not currently clear that there is one correct approach. (Duan N, Manning WG, Jr., Morris CN, Newhouse JP. A Comparison of Alternative Models of Demand for Medical Care. R-2754-HHS. Santa Monica, CA: RAND, 1982.

Mullahy J. Much ado about two: reconsidering retransformation and the two-part model in health econometrics. J Health Econ 1998; 17(3):247-281.

Blough DK, Madden CW, Hornbrook MC. Modeling risk using generalized linear models. J Health Econ 1999; 18(2):153-171.

Diehr P, Yanez D, Ash A, Hornbrook M, Lin DY. Methods for analyzing health care utilization and costs. Annual Review of Public Health 1999; 20:125-144.

Etzioni RD, Feuer EJ, Sullivan SD, Lin D, Hu C, Ramsey SD. On the use of survival analysis techniques to estimate medical care costs. J Health Econ 1999; 18(3):365-380.

Hadley J, Holahan J. Covering The Uninsured: How Much Would It Cost? Health Affairs.06/04/2003.Available online at http://www.healthaffairs.org/WebExclusives/Hadley_Web_Excl_060403.htm.Accessed 08/06/2003)

We examined three alternative modelling approaches: ordinary least squares (OLS), generalized linear modelling (GLM) using a gamma distribution and log link, and a two-part model, using logistic regression to model use/non-use and a generalized linear model using a gamma distribution and log link to model amount of use contingent on any use. In this last approach the predicted probability of use is multiplied by the predicted amount of use contingent on some use to derive a predicted use. The three models produced very similar results, so we decided to use the approach yielding the highest predicted expenditures for the uninsured were they to have insurance coverage. This choice was made to be conservative from the perspective of the CEA.

In our cost-effectiveness models we excluded one person in the Medicare only group, age 66-69 years, whose total expenditures were >$100,000. Had that person been included, the mean expenditures for that group would have been higher for those without private insurance than predicted expenditures with insurance (see Table 8).

We calculated the administrative component of providing insurance as follows. First, the mean amount paid by insurance companies (insurance benefits) for the two privately insured cohorts was obtained from MEPS. We derived the ratio of insurance benefit to total expenditures for the two groups, and applied those ratios to the predicted expenditures for the uninsured cohorts. We assumed that these ratios would be similar for both the insured and uninsured cohorts, if the latter were to obtain insurance. We used the benefit to premium ratio obtained from 1996 National Health Accounts (NHA) data[3] to derive a predicted administrative cost of providing insurance to the uninsured. This process involved a number of assumptions and simplifications. We ignored that some of those imputed administrative costs were profits, reasoning that might approximately offset employer and individual costs associated with insurance. We did this because we were unable to

generate reliable information to derive those separate costs. Because the uncertainties are relatively small compared with the overall expenditures we do not think these simplifications were problematic. We also conducted sensitivity analyses around our expenditure estimates to address this issue. Finally, the fixed and variable costs of providing insurance to such a large number of persons are uncertain. We reasoned that, on average, the administrative costs would largely be related to the predicted utilization, so we apportioned the administrative component in proportion to the predicted total expenditures.

We also calculated per event expenditures for the privately insured and uninsured (Medicare only) groups. We used events in 25 categories provided by MEPS, and for each ratio divided the mean total expenditures in that category for the insurance group by the number of events for persons in that insurance group reporting at least one event. For selected comparisons we examined whether the differences in per event expenditures were statistically significant. We used linear regression models with the per event expenditure as the dependent variable and insurance status as the key independent variable. We adjusted for age, sex, race/ethnicity, income, education, employment status, marital status, region of country, rural vs. urban location, and self-rated health.

<u>Results</u>

There were 8,481 persons < 65 with either no insurance throughout the year or with private insurance. During any month an additional approximately 2% of persons (10% of those uninsured) were without insurance. There were 1,577 persons >65 with Medicare only or Medicare plus private insurance. The distribution of variables by insurance status is shown in Table 6. The relationships between insurance status and sociodemographics are broadly similar to those observed in the 1993 NHIS sample.

Figure 1 shows the actual and predicted expenditures by age-group and insurance status. It can be seen that the three prediction model (OLS, GLM one-part, and GLM two-part) produced very similar results. As shown in Table 7 the mean predicted expenditures using the one-part GLM (gamma distribution with log link) were the highest. Thus we used this method in our CEA. For both groups without private insurance (<65, and >65) their mean expenditures predicted if they were privately insured were slightly higher than those observed for the currently insured cohort, largely because of their lower socio-economic status and lower health status.[2]

Table 8 shows the predicted expenditures by age-group using the one-part GLM and the mean private insurance benefits of the two privately insured cohorts (i.e. < 65 and >65. Despite the differences in overall expenditures the private insurance benefits in the two age-groups are relatively similar. The average insurance benefit to total expenditure ratio for those < 65 was 73.7 %, and 25.8 % for those > 65. The benefit to premium ratio, from 1996 NHA data was 86.7 %. Thus, we derived an average administrative cost of 9.8 % of predicted expenditures for those <65 and 3.4 % for those > 65. In the model, we simply estimated costs at 10% for the 25-65 cohort and 5% for the 65 cohort.

Table 9 shows the per event expenditures for those with and without insurance (or supplemental insurance). For those under 65, per event expenditures were lower for the uninsured, for nearly all categories. The exceptions were for inpatient expenditures and dental expenditures. For inpatient expenditures the reversal reflected one outlier. For both dental and inpatient expenditures the differences were not statistically significant after adjustment for sociodemographics and health status. Several of the other differences showing lower per event expenditures for the uninsured were statistically significant after adjustment. For example, per event outpatient provider visit expenditures were $29.9 (standard error = 4.69) lower for those

without insurance. For those > 65 there were few differences in per event expenditures by insurance status, and none were statistically significant.

C: Decision Analysis Models

Methods and Results

In this section, we present the methods and results together, since model outputs assist the reader in understanding the sensitivity analyses and the overall functioning of the model. We constructed Markov models evaluate changes in expenditures and HRQL in 1-year increments using DATAPRO 4.0 (see Figure 2). In this program, cycles are tracked using a variable termed "_stage" which is recursively set to _stage = _stage + 1 at each cycle. Each model used the age-specific cost and HRQL data listed in Table 4 and Table 8. Tabular values were read as a function of the subjects' age, which was set as Age = _stage + X, where X is equal to 25 or 65 at the start of the analysis, depending on the cohort under study. Ten-year intervals were used in the 25 to 64 cohort and 5-year intervals were used thereafter. Values between intervals were interpolated using the program's built in linear interpolation function.[4]

In the 25-64 cohort model represented in Figure 2, If/Else statements were used to terminate insurance effects at age 65. After this point, it was assumed that the effects of insurance would disappear and all subjects would revert to mortality, HRQL, and expenditure values of the insured. Though lingering effects from remaining uninsured are likely, we had no data on such effects and we took this step to bias the results in favor of the "no insurance" arm of the analysis.

We used tabular, age-specific mortality data. Therefore, in the 25-64 cohort, the model was

allowed to run until virtually all subjects were dead, thus calculating the approximate health-adjusted life expectancy (HALE) of each cohort. In the 25-64 year-old cohort, the termination condition was set to age 80, which is the approximate life expectancy at age 25. In the >65 cohort, the model was set to terminate at 92 years of age, which is the approximate life expectancy in the final age interval (75-85) of the analysis. The model was half-cycle corrected.

As subjects in the decision analysis model age, they are exposed to increased risk of death, decreasing HRQL, and increasing medical expenditures. Both HRQL values and cost values are discounted using the formula:

$$\sum_{1}^{T} \frac{M_t}{(1+d)^{t-1}}$$

where, T = the life expectancy of the cohort, M is the measure being discounted at time t, and d is the discount rate. Those that die exit the cohort and incur no further costs. Therefore, the increased rate of premature death in the uninsured cohort results in increasingly lower costs for this group.

Both 1-way and Monte Carlo Analyses were performed. Since there were a small number of variables in our model, we performed 1-way analyses on all variables. To calculate error in the expenditure estimate for the uninsured cohort, we multiplied age-specific values by a variable that was assigned a value between 0.5 and 1.5 (see Figure 3). We used a broad interval since expenditure estimates were subject to error introduced when patients do not pay for medical care (and expenditures are assigned a value of zero) and when they are billed for charges, rather than negotiated rates.[5] Other error in expenditure estimates includes sampling error, excluded relevant medical costs (such at over-the-counter medications), and various imputations used in generating the MEPS survey. Therefore, we varied expenditures for the insured cohort over a narrower interval of 0.75 to 1.25 (see Figure 4).

On average, age-specific HRQL scores differed only by 4% (see Table 4). To vary the

scores by the percentage difference between the scores results in a percentage of HRQL values that are lower for the insured than for the uninsured, which we felt to be implausible. Even when scores are varied by 3%, the ICER is increased to 120,000 based on lower HRQL among the insured cohort (see Figure 5). Therefore, we varied the HRQL scores by +/- 25% of the predicted difference between the scores (see Figure 6). We felt this more realistically represents the true error in these values.[6]

Varying the hazard ratio between earlier published values[7,8] and the highest plausible value based on our linear regression analyses produced a curvilinear effect on ICERs (see Figure 7). This demonstrates the increasing importance of cost as the subjects in the uninsured arm die off. We did not anticipate any two-way interactions between variables.

In the Monte Carlo simulation, we chose to employ the triangular distribution. This distribution utilizes a baseline value and a high and low value. Points between the baseline value and each extreme are linearly interpolated. We also tested these values using uniform distributions and tested random error using normal distributions and standard error for comparison.

In the Monte Carlo simulation, the distributions are randomly sampled and held constant. One hundred are then entered into a trial using this fixed probability distribution sample. This process is then repeated 10,000 times.[9] When triangular distributions are sampled many times, an approximately normal distribution results (see Figure 8). Our distribution was right skewed due to the asymmetry of the hazard ratio. This process also allows for the calculation of a 95% “credible” interval around these values that is based upon the estimates of random and non-random error in the analysis (see Figure 9). Figure 10 represents the 95% credible ellipsoid for the Medicare plus supplemental insurance cohort.

The model was validated using a life table constructed on a spreadsheet. Table 10 represents

the table used to validate the 25-64 year-old cohort. In this life table, person-years are quality adjusted using the age-specific HALex values we generated from the NHIS. This abridged table was based on average mortality rates over 10 year intervals and values are not discounted; it was therefore necessary to set the discount rate to zero. When this is accomplished incremental life-expectancy values and incremental quality-adjusted life expectancy values are similar between the model and the spreadsheet, differing by 0.1 in life expectancy and 0.2 in QALE. Details of the construction of such tables using the HALex are described elsewhere.[10]

Table 1: Distribution of variables by insurance status in 1993 National Health Interview Survey

	< 65				> 65			
	No Insurance		Insured		No Suppl.		Supplemental	
	N	(%)	N	(%)	N	(%)	N	(%)
Age Group								
25-34	1811	(42.92)	5673	(28.91)				
35-44	1241	(27.72)	6494	(32.15)				
45-54	801	(17.26)	4825	(23.26)				
55-64	545	(12.10)	3188	(15.68)				
66-74					457	(50.78)	2706	(57.77)
75 and older					422	(49.22)	1873	(42.23)
Family Income								
Under $5,000	210	(4.60)	103	(0.53)	36	(4.08)	59	(1.33)
$5,000-$6,999	170	(3.73)	48	(0.24)	57	(6.36)	106	(2.21)
$7,000-$9,999	315	(7.06)	149	(0.71)	122	(13.35)	311	(6.56)
$10,000-$14,999	609	(13.56)	507	(2.46)	140	(16.16)	600	(13.08)
$15,000-$19,999	602	(14.10)	1103	(5.35)	94	(10.95)	582	(12.53)
$20,000-$24,999	420	(9.48)	1441	(7.26)	60	(6.78)	543	(11.91)
$25,000-$34,999	536	(12.36)	3262	(16.10)	69	(7.64)	637	(14.25)
$35,000-$49,999	350	(8.03)	4664	(23.06)	41	(4.49)	421	(9.11)
$50,000 or more	324	(7.60)	6760	(33.73)	41	(5.11)	421	(9.52)
Unknown	862	(19.48)	2143	(10.55)	219	(25.07)	899	(19.51)
Gender								
Male	2213	(52.04)	9648	(49.05)	332	(39.75)	1863	(42.28)
Female	2185	(47.96)	10532	(50.95)	547	(60.25)	2716	(57.72)

Highest Education (Years)				
None	8 (0.18)	5 (0.03)	5 (0.57)	7 (0.20)
1-8 years	282 (6.45)	203 (1.03)	184 (21.00)	466 (9.96)
9-11 years	550 (12.17)	702 (3.38)	157 (17.10)	489 (10.54)
12 years	1806 (41.12)	6363 (31.17)	276 (31.90)	1752 (38.08)
1-3 years	1010 (22.97)	5227 (26.02)	127 (14.63)	814 (17.95)
4 years	447 (10.34)	3935 (19.63)	71 (8.16)	521 (11.60)
5+ years	288 (6.62)	3728 (18.65)	53 (5.95)	519 (11.48)
Unknown	7 (0.16)	17 (0.08)	6 (0.69)	11 (0.20)
Race/Ethnicity				
White	2673 (62.75)	16473 (82.11)	583 (69.92)	4193 (92.46)
Black	768 (15.16)	1852 (8.28)	208 (19.39)	261 (4.57)
Other	198 (4.67)	754 (3.94)	17 (2.46)	44 (1.07)
Hispanic	759 (17.42)	1101 (5.68)	68 (8.23)	71 (1.91)
Work Status				
Employed	3104 (83.83)	16917 (84.03)	72 (8.50)	601 (13.20)
Unemployed	241 (2.21)	446 (2.27)	3 (0.30)	20 (0.43)
Not in Workforce	1053 (13.96)	2817 (13.70)	804 (91.20)	3958 (86.37)
Marital Status				
Married,spouse home	2587 (78.55)	15852 (78.61)	373 (42.69)	2697 (59.51)
Married,spouse not home	73 (0.72)	145 (0.71)	8 (0.88)	40 (0.88)
Widowed	149 (1.71)	346 (1.65)	401 (45.87)	1452 (31.30)
Divorced	582 (7.54)	1521 (7.44)	57 (6.14)	204 (4.24)
Separated	154 (1.70)	344 (1.64)	2 (0.22)	25 (0.56)
Never married	845 (9.67)	1951 (9.85)	38 (4.21)	160 (3.49)
Unknown	8 (0.10)	21 (0.10)	0 (0)	1 (0.02)

Region				
Northeast	687 (15.34)	4294 (20.74)	160 (17.82)	1042 (22.19)
Midwest	716 (15.78)	5524 (26.92)	189 (20.71)	1325 (27.93)
South	1903 (44.43)	6028 (30.65)	346 (40.39)	1408 (32.11)
West	1092 (24.44)	4334 (21.69)	184 (21.08)	804 (17.77)
MSA of Residence				
MSA,Central City	1624 (35.80)	5285 (25.90)	331 (34.24)	1251 (27.33)
MSA,Not Central City	1735 (41.24)	10393 (52.84)	327 (39.88)	2055 (46.51)
Non-MSA,Nonfarm	999 (22.16)	4196 (19.87)	217 (25.38)	1176 (24.10)
Non-MSA,Farm	40 (0.80)	306 (1.39)	4 (0.50)	97 (2.06)
Smoking Status				
Ever Smoker	1243 (58.19)	4909 (49.66)	217 (46.54)	1351 (48.52)
Never Smoker	898 (41.81)	4968 (50.34)	263 (53.46)	1441 (51.48)
Front Seat Belt Use				
Always/Mostly	1459 (68.65)	7904 (80.40)	337 (73.81)	2295 (83.55)
Less Often	648 (31.35)	1933 (19.60)	125 (26.19)	457 (16.45)

Notes: Percentages are adjusted for sampling weights to be nationally representative.

Table 2: Adjusted (logistic regression) risk of no insurance (no supplemental insurance)

Risk Factors	Model I - <65 Beta	Model I - <65 SE Beta	Model II - > 65 Beta	Model II - > 65 SE Beta
Age Group				
25-34	0.9601	(0.1065)		
35-44	0.6751	(0.1067)		
45-54	0.6392	(0.1190)		
55-64	0.0000	(0.0000)		
66-74			0.0675	(0.1124)
75 and older			0.0000	(0.0000)
Family Income				
Under $5,000	1.3480	(0.1895)	0.5030	(0.3200)
$5,000-$6,999	1.8413	(0.2248)	0.6100	(0.2478)
$7,000-$9,999	1.3270	(0.1600)	0.3256	(0.1912)
$10,000-$14,999	0.7936	(0.1024)	-0.1717	(0.1675)
$15,000-$19,999	-0.0087	(0.1051)	-0.3122	(0.2102)
$20,000-$24,999	-0.4750	(0.1055)	-0.9144	(0.2637)
$25,000-$34,999	-0.9923	(0.0967)	-0.9109	(0.2544)
$35,000-$49,999	-1.6916	(0.1273)	-1.3077	(0.3709)
$50,000 or more	-1.9366	(0.1136)	-1.7168	(0.3862)
Unknown	0.0000	(0.0000)	-0.0000	(0.0000)
Female Sex	-0.4164	(0.0607)	-0.3721	(0.1394)
Highest Education (years)				
None	1.1160	(1.2865)	-1.4041	(1.0097)
1-8 years	1.9203	(1.0364)	-0.5949	(0.5999)
9-11 years	1.7584	(1.0165)	-0.9238	(0.5780)
12 years	1.3559	(1.0173)	-1.1878	(0.5899)

1-3 years	1.2849 (1.0156)	-1.2452 (0.6027)
4 years	1.0340 (1.0181)	-1.1447 (0.6718)
5+ years	0.6997 (1.0030)	-1.2677 (0.6613)
Unknown	0.0000 (0.0000)	0.0000 (0.0000)
Race/Ethnicity		
White	-0.4532 (0.1047)	-1.4613 (0.3985)
Black	-0.4846 (0.1205)	-0.0908 (0.4250)
Other	-0.4099 (0.2278)	-0.6143 (0.6406)
Hispanic	0.0000 (0.0000)	-0.0000 (0.0000)
Family Size	0.1754 (0.0274)	0.5070 (0.0950)
Work Status		
Employed	-0.6995 (0.0892)	-0.3726 (0.2029)
Unemployed	-0.2359 (0.1546)	
Not in Workforce	0.0000 (0.0000)	0.0000 (0.0000)
Marital Status		
Married,spouse home	-1.0118 (0.6910)	-0.6925 (0.2920)
Married,spouse not home	-0.3936 (0.7333)	-0.4115 (0.5825)
Widowed	-0.5713 (0.7112)	0.1908 (0.2628)
Divorced	-0.4308 (0.7109)	0.2445 (0.3362)
Separated	-0.9417 (0.7275)	-2.3508 (1.0768)
Never married	-0.5061 (0.7067)	0.0000 (0.0000)
Unknown	0.0000 (0.0000)	
Number of Conditions	-0.0387 (0.0251)	-0.0264 (0.0306)

Region		
Northeast	-0.3198 (0.0965)	-0.8303 (0.2092)
Midwest	-0.7369 (0.0793)	-0.8900 (0.2060)
South	-0.0120 (0.0790)	-0.3276 (0.1741)
West	0.0000 (0.0000)	0.0000 (0.0000)
MSA of Residence		
MSA,Central City	0.3203 (0.2686)	0.2700 (0.6141)
MSA,Not Central City	0.0602 (0.2630)	0.4495 (0.6083)
Non-MSA,Nonfarm	0.1211 (0.2618)	0.4522 (0.6088)
Non-MSA,Farm	0.0000 (0.0000)	0.0000 (0.0000)
Ever Smoker (vs. never)	-0.3119 (0.0655)	0.0163 (0.1520)
Seatbelt (Always vs. less)	-0.3475 (0.0668)	-0.5509 (0.1282)
N insured/supplemental	9814	4579
N uninsured/ no supplemental	2101	879

Notes: Odds Ratios may be obtained by exponentiating the beta coefficients; 95% confidence intervals by adding or subtracting 1.96 times the standard error from the beta coefficient and exponentiating the result. Beta coefficients of 0 reflect the reference group for other categories.

Table 3: Parameter estimates for regressions of health status onto significant covariates for the insured (<65) and those with supplemental insurance (>65).

	Model I - <65		Model II - > 65	
Risk Factors	Beta	SE Beta	Beta	SE Beta
Intercept	0.7033	(0.1106)	0.8281	(0.0850)
Age Group				
25-34	0.0365	(0.0044)		
35-44	0.0235	(0.0043)		
45-54	0.0034	(0.0051)		
55-64	0.0000	(0.0000)		
Age (per year)			-0.0030	(0.0006)
Family Income				
Under $5,000	0.0237	(0.0189)	-0.0221	(0.0256)
$5,000-$6,999	-0.0425	(0.0248)	-0.0058	(0.0228)
$7,000-$9,999	-0.0250	(0.0162)	-0.0145	(0.0147)
$10,000-$14,999	-0.0001	(0.0099)	0.0014	(0.0116)
$15,000-$19,999	0.0021	(0.0077)	0.0010	(0.0135)
$20,000-$24,999	0.0114	(0.0064)	0.0089	(0.0123)
$25,000-$34,999	0.0154	(0.0052)	0.0260	(0.0124)
$35,000-$49,999	0.0191	(0.0055)	0.0389	(0.0118)
$50,000 or more	0.0266	(0.0050)	0.0317	(0.0128)
Unknown	0.0000	(0.0000)	0.0000	(0.0000)
Female Sex	--	--	--	--
Highest Education (years)				
None	0.0846	(0.0848)	0.0216	(0.1628)
1-8 years	0.0725	(0.0787)	-0.0142	(0.0257)
9-11 years	0.0999	(0.0771)	0.0213	(0.0256)

12 years	0.1323	(0.0778)	0.0419	(0.0244)
1-3 years	0.1438	(0.0779)	0.0474	(0.0259)
4 years	0.1595	(0.0776)	0.0750	(0.0267)
5+ years	0.1634	(0.0770)	0.0658	(0.0270)
Unknown	0.0000	(0.0000)	0.0000	(0.0000)
Race/Ethnicity				
White	0.0274	(0.0064)	--	--
Black	-0.0097	(0.0080)	--	--
Other	0.0001	(0.0081)	--	--
Hispanic	0.0000	(0.0000)	--	--
Family Size	--	--	-0.0320	(0.0079)
Work Status				
Employed	0.0482	(0.0048)	0.0432	(0.0088)
Unemployed	0.0461	(0.0081)	0.0623	(0.0331)
Not in Workforce	0.0000	(0.0000)	0.0000	(0.0000
Marital Status				
Married,spouse home	-0.0661	(0.0459)	0.0537	(0.0161)
Married,spouse not home	-0.0370	(0.0473)	-0.0311	(0.0394)
Widowed	-0.0574	(0.0473)	0.0593	(0.0133)
Divorced	-0.0422	(0.0462)	0.0459	(0.0188)
Separated	-0.0558	(0.0478)	0.0697	(0.0238)
Never married	-0.0666	(0.0469)	0.0350	(0.0200)
Unknown	0.0000	(0.0000)	0.0000	(0.0000)
Number of Conditions	-0.0605	(0.0015)	-0.0697	(0.0024)
Region				
Northeast	--	--	-0.0047	(0.0110)
Midwest	--	--	-0.0115	(0.0108)
South	--	--	-0.0230	(0.0107)
West	--	--	0.0000	(0.0000)

MSA of Residence				
MSA,Central City	0.0109	(0.0121)	--	--
MSA,Not Central City	0.0048	(0.0119)	--	--
Non-MSA,Nonfarm	0.0012	(0.0120)	--	--
Non-MSA,Farm	0.0000	(0.0000)	--	--
Current Smoking Status				
Never smoked	0.0070	(0.0140)	0.0191	(0.0415)
Current smoker	-0.0117	(0.0141)	-0.0200	(0.0410)
Former smoker	0.0008	(0.0142)	0.0025	(0.0410)
Smoker, unknown status	-0.0639	(0.0431)	0.0212	(0.0463)
Unknown	0.0000	(0.0000)	0.0000	(0.0000)
Front Seatbelt use				
All or most of time	0.0477	(0.0765)	0.2340	(0.0455)
Some of the time	0.0379	(0.0767)	0.2228	(0.0470)
Once in a while	0.0443	(0.0770)	0.2020	(0.0490)
Never	0.0226	(0.0769)	0.1799	(0.0447)
Don't ride in front	0.0529	(0.0773)	0.1434	(0.0783)
Don't ride in car	0.0058	(0.0839)	0.0404	(0.0590)
Not asked	0.0555	(0.0773)	0.1828	(0.0606)
Refused, don't know	0.0000	(0.0000)	0.0000	(0.0000)

Table 4: Mean health status, actual and predicted, and the probability of mortality by age and insurance status.

	Health-Related Quality of Life			Probability of death	
Age	Insured/ Supplemental	No Insurance/No Supplemental	Predicted*	Insured/ Supplemental	No Insurance/ No Supplemental
25-34	0.9178	0.8838	0.882	0.00055	0.000913
35-44	0.9037	0.8406	0.861	0.001	0.00166
45-54	0.8708	0.7943	0.8251	0.0027	0.004482
55-64	0.8365	0.7254	0.7782	0.00595	0.009877
65-69	0.8069	0.7545	0.7586	0.0161	0.02415
70-74	0.7908	0.7134	0.7402	0.02355	0.035325
75-79	0.739	0.7193	0.7251	0.02885	0.043275
80-84	0.7281	0.6988	0.6988	0.04635	0.069525
>85	0.6754	0.58	0.6734	0.07865	0.117975

*Predicted is the health status of the uninsured cohort, predicted on the basis of their sociodemographic, condition, and behavioral risk factor characteristics. These values were generated using the parameter estimates of the effects of these variables on health status in the insured, shown in Table 3.

Table 5: Adjusted relationship between insurance status and subsequent mortality (Proportional Hazards Model)

	Model I - <65		Model II - > 65	
Independent Variables	Beta	SE Beta	Beta	SE Beta
<u>Age Group</u>				
25-34	-2.0838	0.4245		
35-44	-1.8436	0.2917		
45-54	-0.9104	0.2223		
55-64	0.0000	0.0000		
66-74			-0.7520	0.0964
75 and older			0.0000	0.0000
No Insurance/No Supplement	0.5499	0.2277	0.4451	0.1300
<u>Family Income</u>				
Under $5,000	-0.2425	0.8024	-0.4527	0.4945
$5,000-$6,999	-0.0560	0.7087	-0.2838	0.2960
$7,000-$9,999	0.5570	0.5161	-0.4141	0.2131
$10,000-$14,999	-0.1712	0.4077	-0.1324	0.1740
$15,000-$19,999	0.0188	0.3569	-0.1889	0.1762
$20,000-$24,999	-0.7593	0.4873	-0.4325	0.2027
$25,000-$34,999	-0.3164	0.3734	-0.5511	0.2124
$35,000-$49,999	-0.2610	0.3215	-0.5928	0.2231
$50,000 or more	-0.5734	0.3391	-0.5128	0.2427
Unknown	0.0000	0.0000	0.0000	0.0000
Female Sex	-0.7459	0.1767	-0.6971	0.0998

Highest Education (years)				
None	-0.7424	0.5796	-0.2683	1.1626
1-8 years	4.9420	0.5918	-0.2903	0.6460
9-11 years	4.0085	0.6121	-0.2235	0.6627
12 years	4.2812	0.5722	-0.1991	0.6479
1-3 years	4.5609	0.5546	-0.2064	0.6547
4 years	4.4456	0.5934	-0.1509	0.6618
5+ years	4.5200	0.6288	-0.0249	0.6514
Unknown	0.0000	0.0000	0.0000	0.0000
Race/Ethnicity				
White	1.0859	0.6009	0.5913	0.3839
Black	1.6467	0.6217	0.2455	0.3971
Other	0.9305	0.8164	-0.9591	1.0845
Hispanic	0.0000	0.0000	0.0000	0.0000
Family Size	0.0157	0.0812	0.0327	0.0667
Work Status				
Employed	-0.1544	0.2285	-0.5093	0.1786
Unemployed	-0.1679	0.5588	0.5032	0.8026
Not in Workforce	0.0000	0.0000	0.0000	0.0000
Marital Status				
Married,spouse home	4.1322	0.3845	6.1863	0.9991
Married,spouse not home	-0.3408	0.3833	7.3259	1.0484
Widowed	4.8081	0.4990	6.3393	0.9881
Divorced	4.2046	0.4724	6.4222	0.9942
Separated	4.1344	0.7108	6.6014	1.2353
Never married	4.5087	0.4075	6.2549	1.0298
Unknown	0.0000	0.0000	0.0000	0.0000
Number of Conditions	0.2617	0.0474	0.1571	0.0230

Region				
Northeast	0.4320	0.2925	0.3476	0.1352
Midwest	0.3321	0.3021	-0.0190	0.1316
South	0.0086	0.2786	0.0143	0.1422
West	0.0000	0.0000	0.0000	0.0000
MSA of Residence				
MSA,Central City	0.2158	0.6853	0.2561	0.4471
MSA,Not Central City	0.2742	0.6927	0.1433	0.4406
Non-MSA,Nonfarm	0.3632	0.6876	0.3258	0.4409
Non-MSA,Farm	0.0000	0.0000	0.0000	0.0000
Total Sample	24578		5445	
Total Dying	140		424	

Notes: Hazard Ratios may be obtained by exponentiating the beta coefficients; 95% confidence intervals by adding or subtracting 1.96 times the standard error from the beta coefficient and exponentiating the result. Beta coefficients of 0 reflect the reference group for other categories.

Table 6: Distribution of variables by Insurance Status in the 1996 Medical Expenditure Panel Survey

	< 65		> 65	
	No Insurance	Insured	Medicare only	Medicare+Suppl
Age Group				
25-34	681 (36.63)	1650 (25.57)		
35-44	547 (32.35)	2204 (33.14)		
45-54	357 (20.03)	1810 (26.62)		
55-64	191 (10.99)	1041 (14.67)		
66-74			242 (47.00)	579 (53.69)
>74			260 (53.00)	496 (46.31)
Gender				
Female	952 (56.55)	3251 (49.18)	211 (40.67)	460 (44.79)
Male	824 (43.45)	3454 (50.82)	291 (59.33)	615 (55.21)
Race/Ethnicity				
Other	78 (5.72)	221 (3.80)	11 (2.33)	11 (1.29)
Black	231 (14.70)	658 (8.45)	70 (11.30)	65 (4.12)
White	806 (56.01)	5012 (81.33)	358 (80.55)	957 (92.35)
Hispanic	661 (23.57)	814 (6.41)	62 (5.82)	42 (2.24)
Federal Poverty Level				
<100%	455 (21.74)	200 (2.24)	85 (10.72)	124 (7.30)
100-124%	150 (7.49)	118 (1.32)	47 (10.86)	59 (5.84)
125-199%	424 (24.62)	567 (7.84)	112 (26.18)	208 (20.85)
200-399%	489 (29.72)	2417 (35.15)	135 (31.31)	337 (38.36)
>399%	258 (16.42)	3403 (53.45)	123 (20.92)	347 (27.65)
Educational Level				
Unknown	14 (0.21)	4 (0.02)	3 (0.36)	1 (0.12)
<12 years	688 (31.40)	636 (7.70)	230 (43.56)	344 (31.96)
12 years	607 (36.05)	2280 (32.96)	148 (30.76)	385 (36.16)

13-15 years	273 (19.06)	1622 (24.23)	68 (14.66)	178 (16.58)
16 years	142 (9.81)	1306 (21.16)	35 (6.80)	81 (7.53)
> 16 years	52 (3.48)	857 (13.92)	18 (3.86)	86 (7.65)
Marital Status				
Unmarried	832 (52.76)	1635 (26.53)	262 (52.24)	421 (38.17)
Married	944 (47.24)	5070 (73.47)	240 (47.76)	654 (61.83)
Employment Status				
Unknown	38 (2.00)	17 (0.26)	1 (0.19)	4 (0.40)
Employed	1233 (72.79)	5880 (87.74)	65 (14.08)	157 (14.28)
Unemployed	98 (6.50)	101 (1.57)	6 (1.27)	10 (1.13)
Not Working	401 (20.01)	706 (10.44)	430 (84.46)	904 (84.19)
Region				
Northeast	277 (16.96)	1412 (20.56)	107 (19.88)	213 (18.26)
Midwest	258 (16.00)	1662 (25.69)	110 (24.09)	300 (27.65)
South	724 (40.28)	2243 (32.79)	166 (34.30)	354 (35.97)
West	517 (26.76)	1388 (20.96)	119 (21.73)	208 (18.12)
Urban/Rural				
Rural	435 (23.44)	1336 (17.37)	103 (18.99)	281 (23.91)
Urban	1341 (76.56)	5369 (82.63)	399 (81.01)	794 (76.09)
Self-Rated Health				
Unknown	90 (0.84)	2 (0.01)	13 (0.83)	0
Excellent	446 (29.65)	2344 (36.22)	103 (20.84)	202 (20.05)
Very Good	434 (26.97)	2345 (35.18)	120 (24.20)	304 (28.15)
Good	530 (28.65)	1558 (22.26)	147 (29.00)	323 (28.87)
Fair	229 (11.26)	394 (5.34)	80 (17.05)	183 (16.72)
Poor	47 (2.64)	62 (0.99)	39 (8.08)	63 (6.22)

Notes: Percentages are adjusted for sampling weights to be nationally representative.

Table 7: Mean predicted expenditures using 3 different modelling approaches.

Prediction Model	Mean Predicted Expenditures (for uninsured/Medicare only were they to have private insurance)	
	<65	>65
Ordinary Least Squares	$1657	$5047
Generalized Linear Models (gamma distribution/log link)		
One part	$1775	$5083
Two part	$1773	$5072

Notes: One part model uses the gamma distribution/log link applied to whole sample. Two part model is the product of predicted probability of use and amount of use contingent on use (using gamma distribution and log link)

Table 8: Mean expenditures, and insurance benefits, actual and predicted by insurance status.

A)< 65

Category	Private Insurance	No Insurance Actual	No Insurance Cohort Predicted (If Private Insurance)
<u>Mean Total</u>			
Expenditures	$1739	$ 686	$1775
Insurance Benefit	$1282	--	$1314
<u>Expenditures</u>			
<u>By Age Group</u>			
25-34	$1169	$ 326	$1193
35-44	$1543	$ 519	$1613
45-54	$1935	$ 742	$2219
55-64	$2820	$2280	$3385

B)> 65

	Medicare+ Private Insurance	Medicare only	Medicare only Cohort Predicted (If Private Insurance)
<u>Mean Total</u>			
Expenditures	$4915	$3956	$5083
Insurance Benefit	$1269	--	$1312
<u>Expenditures</u>			
<u>By Age Group</u>			
66-69	$3843	$2788	$4062
70-74	$4145	$2537	$4126
75-79	$5577	$3465	$5682
≥80	$6131	$4835	$6099

Notes: This analysis excludes two persons with actual expenditures > $100,000, with those persons included, mean expenditures for Medicare only group, = $5605 (age 66-69), and $6305 for ≥ 80 year group with private insurance.

Table 9: Per event expenditures by insurance status

Category	<65				>65			
	Insured		Uninsured		Suppl.		Medicare only	
	N	$/event	N	$/event	N	$/event	N	$/event
OFFICE-BASED PROVIDER VISIT EXPENDITURES	4986	100.4	735	72.7	989	95.0	414	85.8
OFFICE-BASED PHYSICIAN VISIT EXPENDITURES	4721	103.6	641	79.5	972	99.4	407	86.4
OFFICE-BASED NON-DR VISIT EXPENDITURES	1922	107.0	245	51.7	478	70.4	147	89.7
OFFICE-BASED CHIRO VISIT EXPENDITURES	294	43.3	49	35.9	38	34.6	11	32.1
OFFICE-BASED NURSE/PRAC VISIT EXPENDITURES	633	67.1	76	32.0	245	47.4	62	35.0
OFFICE-BASED OPTOMTRIST VISIT EXPENDITURES	301	133.1	28	75.9	58	95.6	16	82.0
OFFICE-BASED PHYS ASST VISIT EXPENDITURES	124	105.5	16	57.2	11	42.5	7	53.9
OFFICE-BASED PT/OC VISIT EXPENDITURES	169	101.0	13	40.3	32	103.3	6	83.9
TOTAL OUTPATIENT FACILITY EXP	1037	515.1	115	343.7	382	546.6	127	338.7
TOTAL OUTPATIENT PROVIDER EXP	1037	225.5	115	194.1	382	158.0	127	140.8
OPD PHYSICIAN VISIT EXPENDITURES - FAC	547	894.3	75	364.6	233	802.6	71	480.7
OPD PHYSICIAN VISIT EXPENDITURES - DR	547	402.5	75	157.7	233	242.4	71	203.6
OPD NON-PHYS VISIT EXPENDITURES - FAC	663	281.7	48	135.2	238	274.2	75	207.2
OPD NON-PHYS VISIT EXPENDITURES - DR	663	113.4	48	44.2	238	83.5	75	63.7
ER FACILITY VISIT EXPENDITURES	658	350.1	181	190.2	155	346.8	68	326.8
ER DOCTOR VISIT EXPENDITURES	658	113.4	181	46.1	155	102.3	68	117.5

Table 9 (Continued).

ZERO-NITE IP STAY EXPENDITURES - FAC	39	1320.4	2	31333.6	13	1695.9	2	1424.6
ZERO-NITE IP STAY EXPENDITURES - DR	39	793.4	2	5014.3	13	890.6	2	317.5
IP HOSP STAY EXPENDITURES - FAC	411	4942.7	43	5839.3	190	6355.4	73	7933.9
IP HOSP STAY EXPENDITURES - DR	411	1216.3	43	470.4	190	785.6	73	658.6
TOTAL DENTAL CARE VISIT EXPENDITURES	3468	134.9	328	140.3	529	129.7	161	134.4
GENERAL DENTAL CARE VISIT EXPENDITURES	3224	133.0	285	152.4	499	114.3	153	136.3
ORTHODONTIST VISIT EXPENDITURES	67	189.1	7	169.2	8	108.0	0	0.0
HOME HEALTH AGENCY VISIT EXPENDITURES	30	84.0	3	26.9	76	145.2	46	95.1
HOME HEALTH NON-AGENCY VISIT EXPENDITURES	4	25.7	2	25.3	23	31.4	6	58.4

Table 10. Abridged life table adjusted for health-related quality of life.

All Persons	Probability of Death	Number at beginning of age interval	Number Dying During interval	Person Years In Interval	Cumulative	Life-Expectancy	HRQL	QA Person-Years	HALYs Remaining	QALE
Insured Cohort										
25-35	0.00275	100000	275	998625	5208603	52.086031	0.882	880787.3	3960953	39.610
35-45	0.005	99725	498.625	994757	4209978	42.215875	0.861	856485.7	3080166	30.887
45-55	0.0135	99226.38	1339.556	985566	3215221	32.402889	0.8251	813190.5	2223680	22.410
55-65	0.02975	97886.82	2912.133	964308	2229655	22.777891	0.7782	750424.1	1410490	14.409
65-75	0.1677	94974.69	15927.25	870111	1265348	13.323	0.7586	660065.9	660065.9	6.950
75+	1	79047.43	79047.43	395237	395237	5	0	0	0	0.000
Uninsured Cohort										
25-35	0.0046	100000	456.5	997717.5	5128594	51.285944	0.8838	881782.7	3810739	38.107
35-45	0.0083	99543.5	826.2111	991304	4130877	41.498208	0.8406	833290.1	2928956	29.424
45-55	0.0224	98717.29	2212.254	976112	3139573	31.803679	0.7943	775325.5	2095666	21.229
55-65	0.0494	96505.03	4765.901	941221	2163461	22.418119	0.7254	682761.6	1320341	13.682
65-75	0.1677	91739.13	15384.65	840468	1222240	13.323	0.7586	637579.1	637579.1	6.950
75+	1.0000	76354.48	76354.48	381772	381772	5	0	0	0	0.000

-----This Page Left Intentionally Blank----

Figure 1: Actual and predicted expenditures by age-group and insurance status

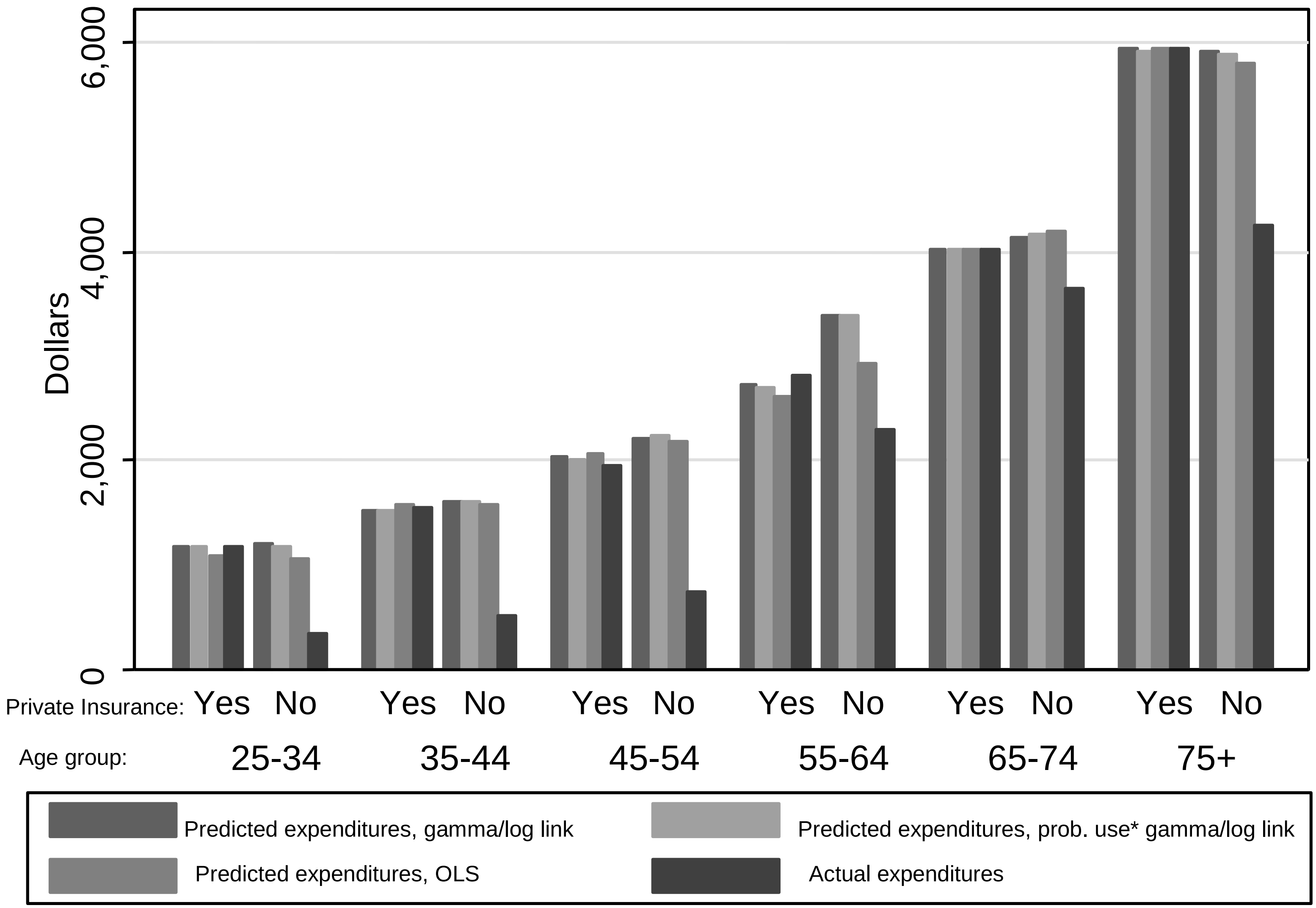

Figure 2. Decision analysis model for the 25-65 cohort.

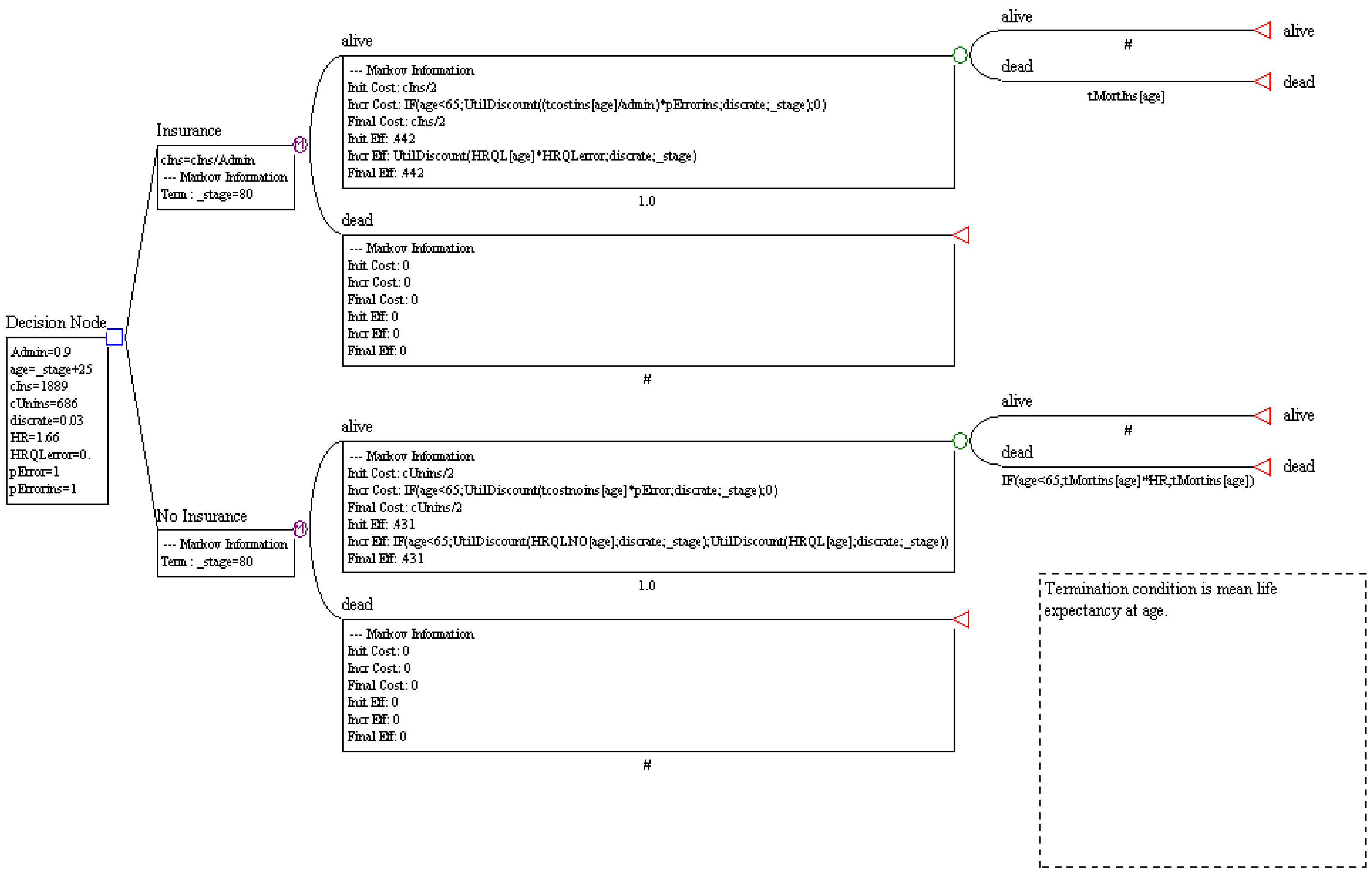

Figure 3. One-way sensitivity analysis on error in the uninsured expenditure estimate for the 25-65 year-old cohort. The variable representing expenditures of the uninsured was multiplied by an error term, which varied between 0.5 and 1.5. The Y-axis indicates the incremental cost effectiveness for different values in the error term.

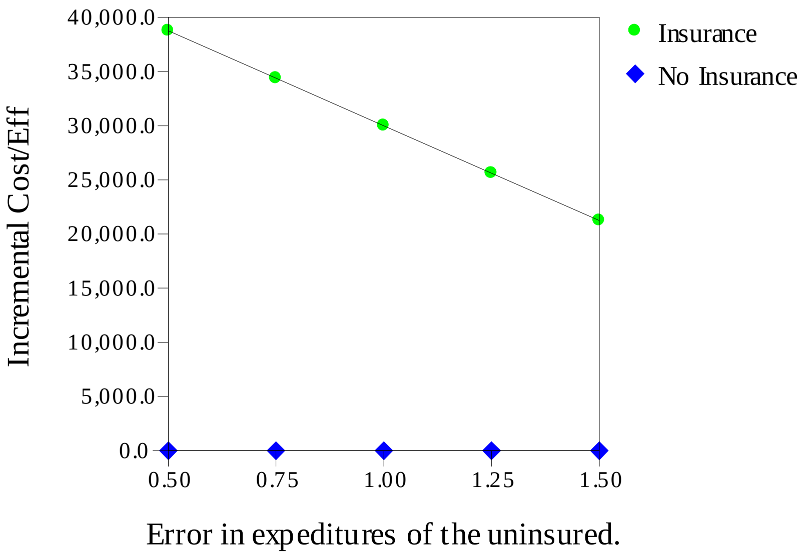

Figure 4. One-way sensitivity analysis on error in the insured expenditure estimate for the 25-65 year-old cohort. The variable representing expenditures of the uninsured was multiplied by an error term, which varied between 0.75 and 1.25. The Y-axis indicates the incremental cost effectiveness for different values in the error term.

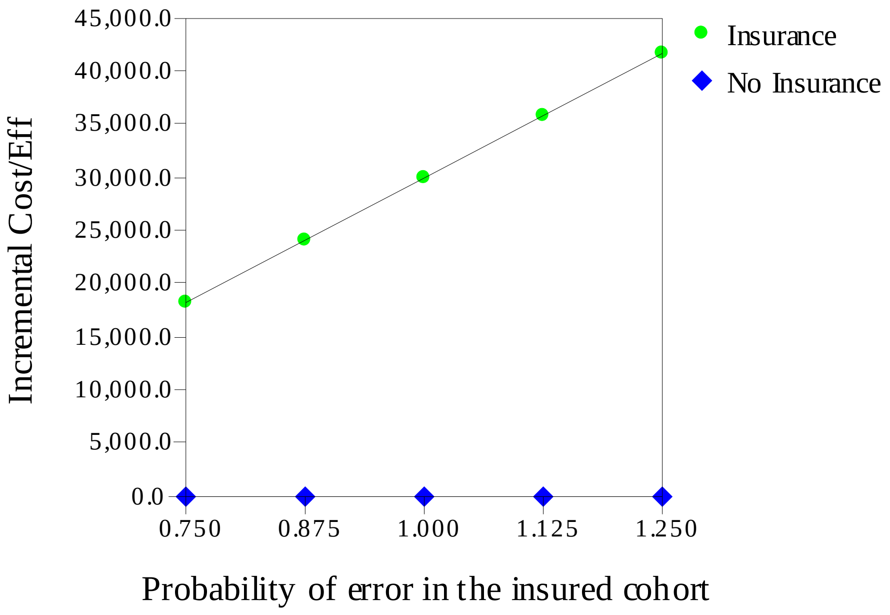

Figure 5. Effect of varying HRQL by 3% in a one-way sensitivity analysis on error in the HRQL estimates for the 25-65 year-old insured cohort.

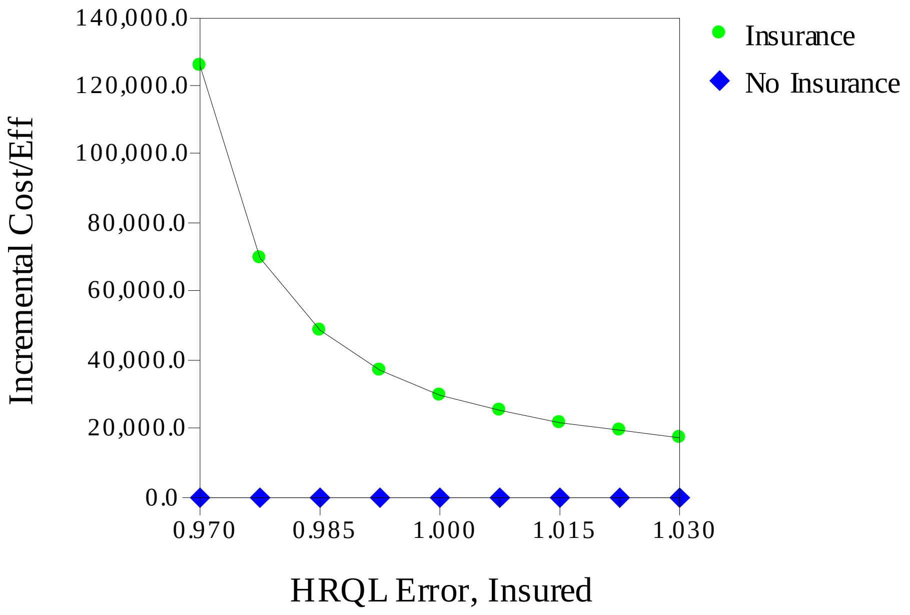

Figure 6. One-way sensitivity analysis on HRQL conducted at 25% of the incremental difference in values between the insured cohort and the uninsured for the 25-65 year-old cohort. The Y-axis indicates the change in overall incremental cost-effectiveness for various values of the hazard ratio.

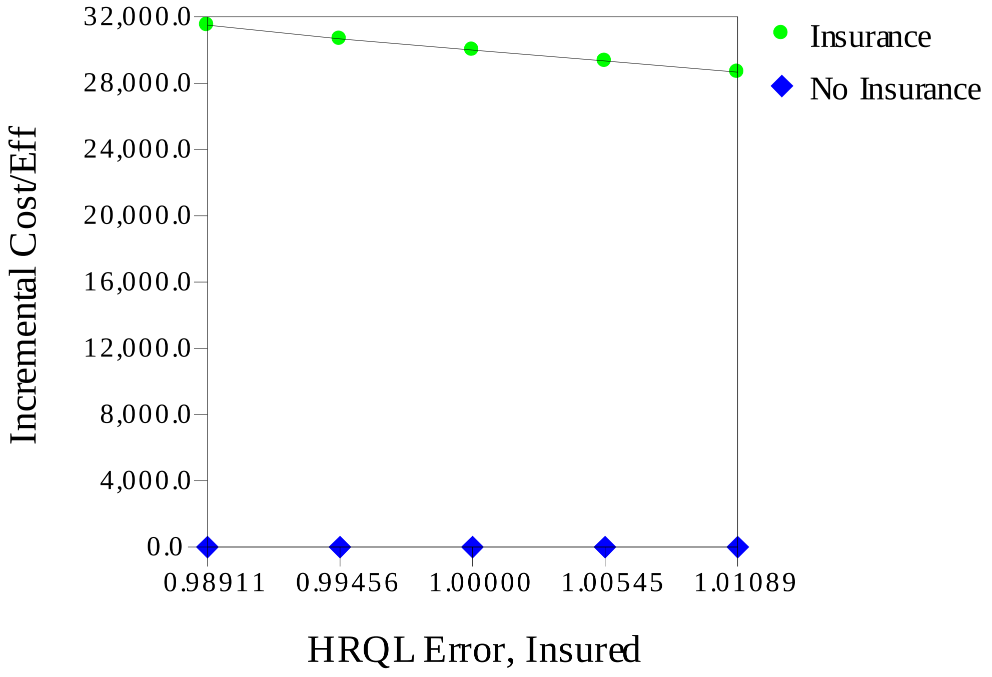

Figure 7. One-way sensitivity analysis on the hazard ratio for the 25-65 year-old cohort. The Y-axis indicates the change in overall

incremental cost-effectiveness for various values of the hazard ratio.

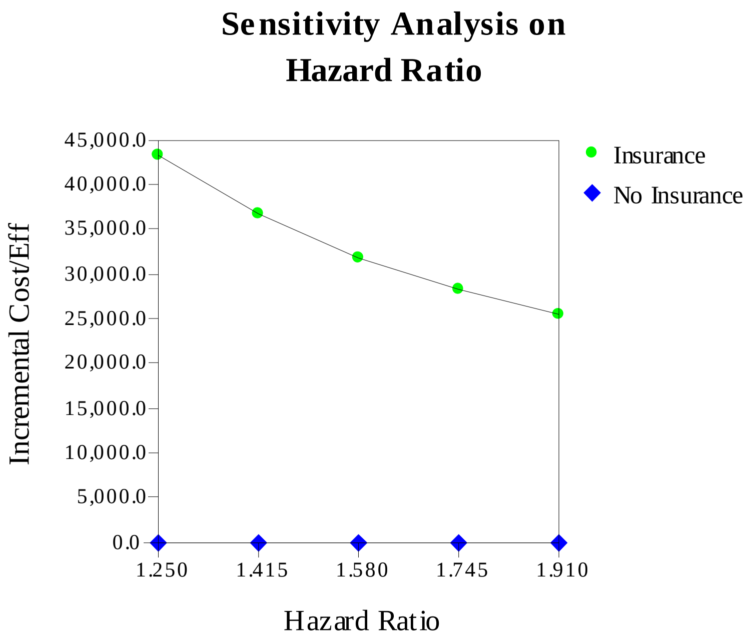

Figure 8. Probability distribution of incremental ICERs for the 25-65 year-old cohort.

ICER
Insurance vs. No Insurance

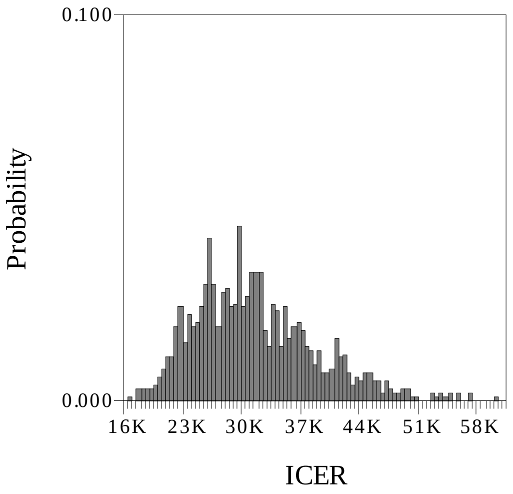

Figure 9. Ninety-five percent credible ellipsoid for the 25-65 year old cohort. In this graph, incremental cost is plotted on the Y-axis and incremental effectiveness is plotted on the X-axis. Points to the left of the dashed line exceed $50,000 per QALY and the interior of the circle contains 95% of all observations.

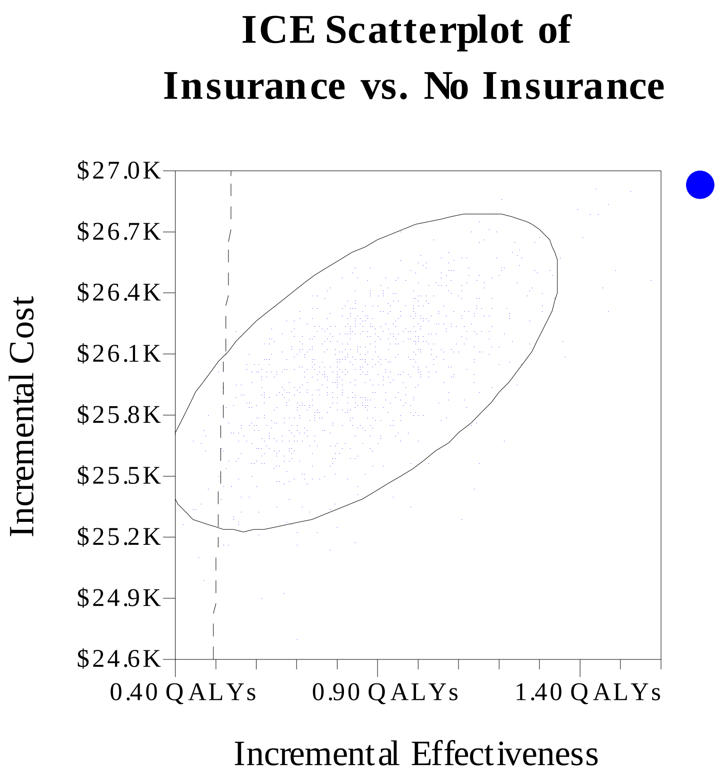

Figure 10. Ninety-five percent credible ellipsoid for the Medicare+supplemental cohort. In this graph, incremental cost is plotted on the Y-axis and incremental effectiveness is plotted on the X-axis. Points to the left of the dashed line exceed $50,000 per QALY.

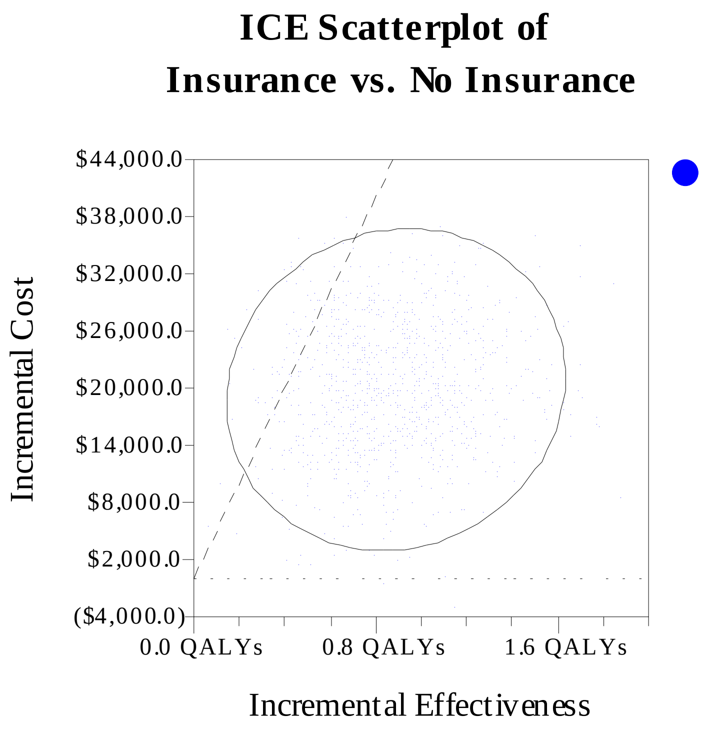

Notes

[1] Smoking status exhibited a complex relationship with insurance status. Current smokers were less likely to be insured. However, former smokers were more likely to be insured, as were never smokers. We reasoned that former smokers, whose health status was lower than current smokers, probably had quit at least in part because of their health problems, and probably influenced by their health care. From the perspective of smoking reflecting possible confounding with the effects health insurance on health (that is, a measure of the possible "healthy choice" bias) we categorized persons as ever smokers vs. never smokers.

[2] Self-rated health (excellent, very good, good, fair, or poor) was used because there were no systematic indicators of morbidity in MEPS. We consider that inclusion of this variable resulted in over-adjustment, since self-rated health itself reflects, in part, the effects of health insurance.

[3] Levit KR, Lazenby HC, Braden BR. National health spending trends in 1996. National Health Accounts Team. Health Aff (Millwood) 1998; 17(1):35-51

[4] Data 4.0 Professional User's Manual. TreeAge Software: Williamstown, MA, 2001.

[5] Medical Expenditure Panel Survey. Agency for Health Research and Quality. Available online at: http://www.meps.ahrq.gov. Accessed 7/8/02.

[6] Baker DW, Sudano JJ, Albert JM, Borawski EA. Lack of health insurance and decline in overall health in late middle age. N Engl J Med 2001;345:1106-12.

[7] Franks P, Clancy CM, Gold MR, Nutting PA. Health insurance and subjective health status: data from the 1987 National Medical Expenditure survey. Am J Public Health 1993;83:1295-9.

[8] Sorlie PD, Johnson NJ, Backlund E, Bradham DD. Mortality in the uninsured compared with that in persons with public and private health insurance. Arch Intern Med 1994;154:2409-16.

[9] Weinstein M, Munink M, Gazelle GS. Representing first- and second-order uncertainties by Monte Carlo simulation for groups of patients. Med Decis Making 2000;20:314:322.

[10] Muennig P, Gold MR. Using the Years-of-Healthy-Life measure to calculate QALYs. Am J Prev Med 2001;20:35-39.
